# A tillering inhibition gene influences root–shoot carbon partitioning and pattern of water use to improve wheat productivity in rainfed environments

**DOI:** 10.1093/jxb/erv457

**Published:** 2015-10-22

**Authors:** P.W. Hendriks, J.A. Kirkegaard, J.M. Lilley, P.J. Gregory, G.J. Rebetzke

**Affiliations:** ^1^CSIRO Agriculture, PO Box 1600, ACT 2601Australia; ^2^ENESAD, Dijon France now Domaine le Pérou, 18170 Le ChateletFrance; ^3^Department of Soil Science, University of Reading, Whiteknights, Reading RG6 6DW, UK

**Keywords:** Drought, phenotyping, root distribution, root length, root weight, *tin*, water use, wheat.

## Abstract

Genetic modification of shoot and root morphology has potential to improve water and nutrient uptake of wheat crops in rainfed environments. Near-isogenic lines (NILs) varying for a tillering inhibition (*tin*) gene and representing multiple genetic backgrounds were phenotyped in contrasting, controlled environments for shoot and root growth. Leaf area, shoot and root biomass were similar until tillering, whereupon reduced tillering in *tin*-containing NILs produced reductions of up to 60% in total leaf area and biomass, and increases in total root length of up to 120% and root biomass to 145%. Together, the root-to-shoot ratio increased two-fold with the *tin* gene. The influence of *tin* on shoot and root growth was greatest in the cv. Banks genetic background, particularly in the biculm-selected NIL, and was typically strongest in cooler environments. A separate de-tillering study confirmed greater root-to-shoot ratios with regular tiller removal in non-*tin*-containing genotypes. In validating these observations in a rainfed field study, the *tin* allele had a negligible effect on seedling growth but was associated with significantly (*P*<0.05) reduced tiller number (–37%), leaf area index (–26%), and spike number (–35%) to reduce plant biomass (–19%) at anthesis. Root biomass, root-to-shoot ratio at early stem elongation, and root depth at maturity were all increased in *tin*-containing NILs. Soil water use was slowed in *tin*-containing NILs, resulting in greater water availability, greater stomatal conductance, cooler canopy temperatures, and maintenance of green leaf area during grain-filling. Together these effects contributed to increases in harvest index and grain yield. In both the controlled and field environments, the *tin* gene was commonly associated with increased root length and biomass, but the significant influence of genetic background and environment suggests careful assessment of *tin*-containing progeny in selection for genotypic increases in root growth.

## Introduction

Wheat crops are commonly grown in rainfed environments where variable rainfall and the occurrence of terminal water deficits limit biomass and grain yield. Developing novel germplasm and/or management practices must take account of the dominant stress development patterns in the target environment (Chenu *et al.*, 2013). In many regions of the world, growth of winter cereals is heavily reliant on stored soil water (Richards *et al.*, 2002; Chenu *et al.*, 2013). Increasing water deficits around early stem elongation can limit tiller production to slow canopy development (Duggan *et al.*, 2005a; Mitchell *et al.*, 2013) while drought immediately prior to and during anthesis can reduce floret fertility to reduce grain number (Saini and Aspinall, 1981). Water limitation during grain-filling reduces assimilate supply to developing grains, reducing final yield and quality (van Herwaarden *et al.*, 1998; Mitchell *et al.*, 2013). As a consequence, the timing and severity of water deficit influence the final impact on grain yield, with some plasticity in yield possible through compensating processes such as changes in tiller or ear growth (Sadras and Rebetzke, 2013).

Rapid leaf area development and early ground cover is a breeding target in rainfed environments where crop water use arises largely from in-crop rainfall (Richards *et al.*, 2002). However, in seasons or environments where water is limiting late in the growing season, excessive early leaf growth can exhaust available soil water prematurely, leaving inadequate moisture to sustain grain growth after anthesis (Passioura, 1976, 1977; Richards and Townley-Smith, 1987). Tiller number is a major determinant of leaf area and canopy development, and, at conventional sowing rates, as many as 15 tillers may be initiated on each plant (Berry *et al.*, 2003; Duggan *et al.*, 2005a). However, up to 75% of these tillers senesce before anthesis even at lower plant densities (Stapper and Fischer, 1990; Berry *et al.*, 2003; Mitchell *et al.*, 2013; Moeller *et al.*, 2014) or where growing conditions are unfavourable (Duggan *et al.*, 2005a, b). The death of tillers represents a loss in water and nutrients that cannot be fully recovered by translocation of stored reserves in well-watered (Berry *et al.*, 2003) and water-limited (Geiger and Fondy, 1980) environments. Therefore, control of excessive tillering may be useful in slowing water use in environments where water is limited and terminal droughts are predominant.

Manual de-tillering of free-tillering wheat genotypes has been shown to slow water use, leaving more water for carbon assimilation during grain-filling (Jones and Kirby, 1977; Islam and Sedgely, 1981). A major tiller inhibition (*tin*) gene can restrict tiller production to as few as one, but more commonly 2–4 tillers per plant (Richards, 1988; Duggan *et al.*, 2005a; Moeller *et al.*, 2014). Early studies comparing wheat lines with and without the *tin* gene reported little difference in leaf area index (LAI) owing to compensatory increases in individual leaf size (Yunusa and Sedgley, 1992; Duggan *et al.*, 2005a). More recently, *tin*-containing near-isogenic lines (NILs) were identified that reduce leaf area and slow water use, thereby increasing grain yield relative to free-tillering NILs under controlled water deficit conditions (Mitchell *et al*., 2012, 2013).

Genetic modification of cereal root systems is developing as a potential breeding objective in wheat varietal development programmes (Lopes and Reynolds, 2010; Wasson *et al*., 2012, 2014). Evidence for genetic potential for root modification is well established in studies targeting improved water and nutrient uptake (e.g. Christopher *et al.*, 2013; Wasson *et al.*, 2014), while field studies have demonstrated that capture of as little as 10mm post-anthesis water from the subsoil can increase wheat grain yield by 0.6 t ha^–1^ under terminal drought (Kirkegaard *et al.*, 2007). The *tin* gene reduces tiller number, but has also been reported to have pleiotropic effects on other plant characteristics including increases in leaf and stem size, and reductions in specific leaf area (SLA) (Richards, 1988; Duggan *et al.*, 2005b). Duggan *et al.* (2005b) also provided some evidence for small increases in the root-to-shoot ratio in *tin*-containing lines, while Mitchell *et al.* (2013) reported increased water use at different soil depths for *tin*-containing NILs. Similarly, wheat lines selected for reduced spike number in the UK had significantly increased water extraction compared with high spike number selections, especially below soil depths of 90cm (Innes *et al.*, 1981). Thus it appears possible that genotypes containing the *tin* gene may simultaneously reduce excessive early above-ground biomass while maintaining or increasing root growth at depth. Characteristics that slow or defer water use without compromising yield potential, while increasing the capacity to access deeper water and soil nutrients during grain filing, are likely to improve wheat performance in storage-driven, rainfed environments.

The aims of this study were to confirm earlier reports of the association of the *tin* gene with an increased root-to-shoot ratio (Duggan *et al.*, 2005b), and establish whether the resulting root and/or shoot changes contributed to improved agronomic performance under rainfed conditions in the field. Regular assessments were made of *tin* NILs (biculm and oligoculm *tin*, and free-tillering, non-*tin*) until mid-booting across a range of air temperatures in controlled glasshouse and outdoor conditions using multiple genetic backgrounds. The contrasting air and soil temperatures were selected to explore the potential impact of genotype×environment interaction and thereby assess the robustness of genic variation for root, shoot, and root-to-shoot partitioning in *tin* NILs. Responses were then assessed through validation of the same NILs in a rainfed field environment targeting greater soil water availability during the grain-filling stage.

## Materials and methods

### Reduced-tillering NILs

The influence of the *tin* reduced-tillering allele was investigated in BC_3_-derived NILs generated in genetically unrelated wheat backgrounds, cvs Banks and Kite (Ө_AB_=0.09, i.e. the probability of alleles at a random locus in Banks and Kite being identical by descent was <10%). The Banks NILs comprised three sister lines, Banks+*tin* ‘biculm’ (hereafter ‘B++’ producing 1–2 tillers per plant), Banks+*tin* ‘oligoculm’ (hereafter ‘B+’, 3–4 tillers per plant), and Banks–*tin* ‘free-tillering’ (hereafter ‘B–‘ with 6–15 tillers per plant) (Moeller *et al.*, 2014). The Kite NILs comprised the Kite+*tin* ‘oligoculm’ (hereafter ‘K+’, 2–3 tillers per plant) and Kite–*tin* ‘free-tillering’ (hereafter ‘K–’ with 6–15 tillers per plant). All reduced-tillering *tin* lines contained the *tin* gene from the reduced-tillering donor, Moroccan wheat landrace ‘492’ (Atsmon *et al.*, 1986). In addition, a BC_1_F_5_-derived, *tin* and non-*tin* pair in the Silverstar genetic background was included in the first year (2003) of assessments. All lines were genotyped to confirm the presence of the *tin* allele (Spielmeyer and Richards, 2004). Separate studies confirm small differences in flowering between NILs of <3 d (Moeller *et al.*, 2014).

### Shoot and root growth under controlled-environment conditions

Studies were conducted in both 2003 and 2004 in cooled and heated glasshouses producing mean temperatures of 13 °C and 20 °C, respectively. An additional cool, outdoor environment (mean temperature of 7 ºC) was included in 2004 to extend the potential range of sampled conditions. All seed were obtained from mother plants grown in the same glasshouse environment to avoid confounding genetic and seed source (maternal environment) effects. Seed were then sized between 40mg and 45mg, and 10 seed were randomly sampled from each line for embryo sizing using a stereomicroscope with a graduated ocular lens.

Seed used for each controlled-environment experiment were sampled from the same growing (glasshouse) environment and sized for consistency to within a range of 45–50mg. The resulting range in average kernel size was commonly small, averaging 3mg within each NIL set (data not shown). Embryo sizes, estimated on these graded seed as embryo (surface) area, were 4.96 (B++), 4.97 (B+), 5.00 (B–), 5.13 (K+), and 5.16 (K–) mm^2^. Differences in embryo area (and length and width; data not shown) were not statistically significant (*P*>0.05) within or between the Banks and Kite genetic backgrounds.

In all experiments, a single wheat plant was grown in 0.5 m long, 90mm diameter tubes for the first three harvests, and 1.5 m long, 90mm diameter PVC tubes for later harvests. A soil mix comprising 75% river sand and 25% potting compost was used in all studies. Adequate nutrients and water were provided to sustain wheat growth, and plants were regularly checked for pests. Genotypes were sown into a randomized complete block design containing three (2003) or four (2004) replicates for each harvest. Tubes were positioned on a regular 20×20cm grid and rotated weekly within each block. In 2003, up to seven samples were collected at regular intervals from 130 °Cd to 923 °Cd (mid-booting) whereas only three samples were collected in 2004 between 232 °Cd and 543 °Cd (early stem elongation). The Silverstar NILs were sampled on only two occasions, at weeks 3 and 7 after sowing.

At each harvest, shoots and roots were separated, and roots then washed from the soil on a 0.5mm mesh. Compost and/or sand were then carefully removed with forceps by close examination of the samples. Measurements were made of the numbers and lengths of seminal and branch roots on each plant using a WinRhizo^®^ root scanner following their separation using a stereo-binocular microscope. Roots were then dried at 70 °C in an air-forced oven for 72h before weighing. Measurements were also made on shoots of the numbers of leaves and tillers (primary, secondary, and coleoptile), and mainstem and tiller leaf areas using a Delta-T^®^ planimeter before drying and weighing as for roots. The root-to-shoot ratio was calculated as root dry weight÷shoot dry weight.

### De-tillering effects on root growth

The free- and reduced-tillering Banks (B–, B+) and Kite (K–, K+) NILs, and the free-tillering variety Seri were seed-sized to a range of 40–45mg. Three seed were sown into 1.5 m long tubes containing the soil mix described above before moving into a brightly lit glasshouse set at temperatures of 15/10 °C (day/night). Tubes were positioned and rotated weekly as described above. Seedlings were thinned to one plant per tube. A random subset of the free-tillering lines were pruned weekly of all tillers (‘detillered’) while the remainder were left unpruned and their tillers allowed to grow. Watering was undertaken every 1–2 d, and nutrients were supplied during growth. Pruning was continued until the first mainstem for a given background reached anthesis, whereupon all tubes for this background were washed free of soil, and roots and shoots were separated and weighed. All line×pruning treatments were replicated four times.

### Shoot and root validation in the field

The Banks and Kite NILs were sown into 10 row (rows spaced 0.18 m apart), 4 m long plots at Bethungra in southern NSW (34.76 °S, 147.85 °E, 297 m altitude). Bethungra is located in the high rainfall wheat zone with average annual rainfall of 530mm. The soil was a Red Kandosol comprising a silty loam for the upper 0.15–0.2 m grading to a pale-coloured medium clay at depth previously described in Kirkegaard *et al.* (2007). Land for this experiment had been an alfalfa (*Medicago sativa* L.) pasture for four years, and was sprayed with the herbicide glyphosate 10 months prior to the experiment, with cultivation (to 100mm depth) undertaken prior to sowing. The experiment was a randomized complete block design with four replicates, and was sown on 8 June 2004 at a target sowing density of 200 seed m^–2^ and sowing depth of 30mm. Two plots of each entry were also sown at a reduced density of 120 seed m^–2^. Nutrients were supplied at sowing as Starter 15^®^ applied at 103kg ha^–1^, but no further N was required due to the previous alfalfa crop. The experiment received natural rainfall, and plots were maintained free of weeds and diseases with the application of appropriate herbicide and fungicide control measures.

Shoots and roots were sampled at 15, 29, 44, 58, and 72 days after sowing (DAS), corresponding to emergence and approximately 1.6, 2.9, 4.2, and 5.2 leaves on the mainstem, respectively. For the first sample, ~10 plants were dug from each plot by hammering and sampling from a metal-sided box 200×180mm in size to a depth of 200mm, and the roots and shoots separated. For samples at 29 and 44 DAS, polyvinyl chloride (PVC) columns 200mm long and 75mm in diameter were pushed into the soil in the row above plants shortly after emergence. Each sample contained one or two plants. For the 58 and 72 DAS sampling, shoots were sampled by cutting plants at ground level from two parallel, 0.5 m long sections of a row. Roots were sampled from the same areas by hammering and sampling with the metal-sided box 200×180mm in size for the upper 200mm, and then immediately below using a 90mm metal tube to a depth of 800mm. Root and shoot measurements were as for the outdoor and phytotron studies above but were expressed on a unit area basis. Deep soil cores (1.8 m long and 42mm ID) were removed from plots (four per plot) using a tractor-mounted hydraulic coring rig at maturity to determine rooting depth for the Banks-derived NIL plots only.

Soil water use was monitored regularly for each plot from sowing using neutron moisture readings with a local calibration (Kirkegaard *et al.*, 2007). Plant development was recorded after Zadoks *et al.* (1974), and at anthesis a 0.18 m^2^ area was sampled in each plot for biomass (both plant densities). Estimates of canopy temperature (after Rebetzke *et al.*, 2013b) and leaf diffusion resistance (later converted to leaf conductance after Rebetzke *et al.*, 2003) were obtained for each plot mid-way through grain-filling. Numbers of green leaves per plant were recorded for five plants in each plot from mid grain-fill onward. At maturity, between 80 and 120 culms were hand-cut at ground level using a 40cm wide quadrat oriented across three-bordered rows. Samples were air-dried at 35 °C for 3 d and weighed before and after threshing, the numbers of fertile spikes counted, and the harvest index calculated as the ratio of grain to total above-ground biomass. Plots were end-trimmed to ~3.6 m length and the outside border rows removed before machine harvesting. Kernel weight was measured for a sample of 200 grains from each harvest index sample, and grain number (m^–2^) was calculated from kernel weight and plot yields. Nitrogen content at anthesis and maturity was determined from N concentrations at anthesis, and in mature grains using near-infrared spectroscopy (after Rebetzke *et al*., 2008*a*).

### Statistical analysis

Each environment was analysed separately, with the best spatial models being determined after first fitting the experimental design and then modelling the residual variation with autoregressive row and column terms. Significant spatial effects were then identified and plot residuals assessed for the need for fitting of other (e.g. linear) effects (Cullis *et al.*, 1996). Specific contrasts reflecting the separate hypothesis of effect of presence or absence of *tin*, effect of tiller expression within *tin* (Banks NILs only), and the interaction of *tin* with genetic background were tested for statistical significance using mixed linear models in the SAS procedure MIXED (SAS, 2013). A second set of analyses were undertaken across environments and sample dates to assess the association for seminal and branch root lengths with total root length and biomass. Variance and covariance components were estimated by the method of restricted maximum likelihood using mixed linear models in the SAS procedure MIXED. Genotypic correlations and their approximate standard errors were then estimated after Holland (2006). Statistical significance under the null hypotheses was reported at a *P*-value (α) of 0.05 unless otherwise indicated.

## Results

### Controlled-environment studies

#### Tiller and leaf area development

**Fig. 1.**
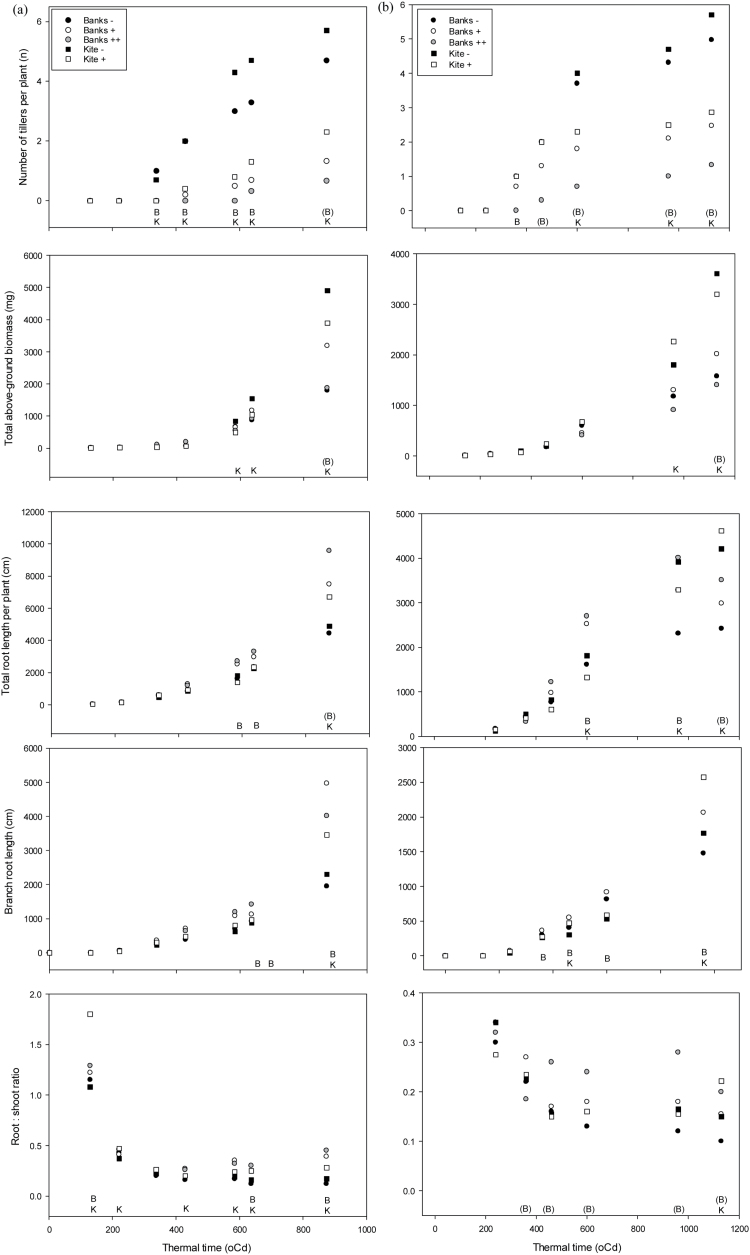
Shoot and root growth for Banks (circles) and Kite (squares) tillering NILs (open symbol=+*tin* allele, shaded = biculm) assessed over multiple sampling dates in 2003 in (a) cool and (b) warm glasshouses. ‘B’ and ‘K’ denote statistical difference between the Banks and Kite *tin* and non-*tin* NILs, respectively, and ‘(B)’ indicates biculm and oligoculm *tin*-containing Banks NILs are statistically different at *P*=0.05.

Tillers first appeared in both *tin* and non-*tin* NILs in Banks and Kite backgrounds at the 3.5-leaf stage (~350 °Cd) in cool and warm environments in both years (e.g. 2003 in Fig. 1; for 2004, see Supplementary Fig. S1 at *JXB* online). At final harvest, up to 10 tillers comprising mainstem, secondary, and occasionally tertiary tillers were observed on individual plants in both Banks and Kite non-*tin* NILs (data not shown). Coleoptile tillers were also commonly observed on plants, and particularly for the Kite genetic background where their frequency was >50% (data not shown). At final harvest, average numbers of tillers per plant for the non-*tin*, B–NIL was 4.6 and 3.9 for cool and warm 2003 environments (Fig. 1), and 4.1 and 3.2 for cool and warm 2004 environments, respectively (Supplementary Fig. S1). The influence of the *tin* gene on tiller reduction at final harvest was greatest for the Banks background and particularly for the biculm B++ (1–2 tillers per plant) and to a lesser extent oligoculm B+ (2–3 tillers per plant) NILs. Together, the reduction in tiller number was ~70% and 60% for B++ in cool and warm environments, and 40 and 25% for B+ in cool and warm environments, respectively. Differences in tiller number and size were small and not statistically significant between the *tin*-containing B++ and B+ NILs (e.g. Fig. 1 and Table 1). The numbers of tillers in the Kite background were commonly fewer for the oligoculm K+ (Fig. 1; Supplementary Fig. S1). In general, statistically significant differences in tiller number between Kite *tin* NILs were delayed until later harvests in warm environments. The influence of *tin* on tillering in the Silverstar genetic background was consistent with tiller reduction observed in the Banks NILs (Table 1).

**Table 1.** Selected shoot and root parameter means for Banks and Kite tiller inhibition NILs measured in cool and warm environments in 2003 and 2004 All environments were sampled at between 380 °Cd and 450 °Cd. Data are also given for Silverstar NILs sampled later at week 7 (585 °Cd and 570 °Cd for cool and warm environments, respectively).

Environment	Entry	No. of leaves	Tillers per plant (*n*)	Tiller leaf area (cm^2^)	Tiller biomass per plant (mg)	Mainstem leaf area (cm^2^)	Total leaf area (cm^2^)	Specific leaf area (cm^2^ g^–1^)	Total shoot weight (mg)	No. of seminals per plant (*n*)
2003 cool	Banks–	4.2	2.0	2.1	69	46	48	439	217	4.7
	Banks+	4.3	0.3*	0.1**	2**	48	48	391*	191*	5.0
	Banks++	4.4	0.0*	0.0**	0**	45	45	354**	192*	5.0
	Kite–	4.2	2.0	1.3	48	48	49	421	213	3.7
	Kite+	4.2	0.4*	0.3**	13**	61**	61*	409	228	3.3
	Silverstar–	5.9	5.3	115	588	127	242	356	1310	4.1
	Silverstar+	6.2	1.7**	32**	118**	124	156**	310**	768**	4.9
2003 warm	Banks–	4.1	2.0	2.3	48	43	45	469	174	5.0
	Banks+	4.3	1.0*	1.2*	28*	44	45	432*	171	4.3*
	Banks++	4.3	0.0*	0.0**	0**	49*	49	362**	175	5.0
	Kite–	4.6	2.0	2.9	67	53	56	431	239	4.3
	Kite+	4.3	1.8	2.3*	54	46*	48*	429	195**	4.7
	Silverstar–	5.8	6.7	77	420	93	170	384	778	4.7
	Silverstar+	6.0	2.7**	84	483*	114*	198*	332*	1005**	5.6
2004 cool	Banks–	4.1	2.0	0.5	73	33	34	374	200	5.3
	Banks+	4.4	1.0*	0.2	71	37	37	328**	237*	5.3
	Banks++	4.2	1.0*	0.0*	12**	38	38	264**	192	4.7*
	Kite–	4.3	2.3	2.8	116	41	44*	334	254	5.3
	Kite+	4.2	2.0	1.9*	81*	49*	51	293*	325**	4.8*
2004 warm	Banks–	3.7	1.7	0.9	28	42	43	491	129	4.3
	Banks+	3.9	0.5*	0.2*	6	44	44	461**	137	4.3
	Banks++	3.8	0.0*	0.1**	4	40	40*	412**	141	4.5
	Kite–	3.8	1.8	0.7	24	50	51	435	159	4.3
	Kite+	3.7	0.8*	0.4	14	47	47*	449	143*	4.0

* and ** denote that restricted-tillering, *tin* NIL means are statistically different from non-*tin*, free-tillering sister NIL means at *P*=0.05 and 0.01, respectively. –, +, ++ refer to free tillering, oligoculm and biculm NILs, respectively.

The *tin* and non-*tin* NILs were not statistically different for leaf area or shoot biomass until soon after commencement of tillering (Fig. 1; Supplementary Fig. S1 at *JXB* online). Shoot morphological differences were subsequently assessed for NILs sampled for all genetic backgrounds at the same approximate developmental stage in all environments (~380–460 °Cd) (Table 1). At this stage, the number of mainstem leaves was similar for NILs and genetic backgrounds. Total leaf areas were commonly greater for the non-*tin* NILs, reflecting their greater tillering and tiller leaf areas. Among different *tin* NILs, the biculm B++ tended to produce smaller total leaf areas, reflecting production of fewer tillers and smaller tiller leaf area (Table 1). The SLAs were generally smaller for *tin*-containing NILs and particularly for the extreme reduced-tillering B++. Numbers of mainstem leaves, leaf areas, and SLA were maintained with later sampling, and were consistent across genetic backgrounds including the Silverstar background (Table 1). Consistent with leaf areas, seedling dry weights were similar for *tin* and non-*tin* NILs until early tillering, where reductions in tiller number and leaf area reduced total plant dry weights for *tin*-containing Banks, Silverstar, and sometimes Kite NILs (Fig. 1). In the Banks background, the B++ NIL was commonly smaller than B+ for total plant dry weight. Over all sampling dates in all controlled-environment experiments, genotypic increases in tiller number were associated with significant increases in total biomass (*r*_g_=0.52, *P* <0.01) and leaf areas (*r*_g_=0.62, *P*<0.01), and reductions in SLA (*r*_g_= –0.71, *P*<0.01). Biomass and leaf area were themselves strongly correlated (*r*_g_=0.87, *P*<0.01). Yet despite these relationships, *tin*-containing wheats tended to produce larger, heavier main stems containing large, thicker leaves to maintain reasonably large total plant dry weights (e.g. Table 1).

#### Root growth

Genotypic differences in total root length were typically small in early harvests, with differentiation between NILs becoming more evident at, or soon after, the first appearance of tillers (Fig. 1; Supplementary Fig. S1 at *JXB* online). At this time, the *tin*-containing NILs produced significantly greater root length in both Banks and Kite genetic backgrounds, and this difference was consistent in both cool and warm environments. The magnitude of difference in *tin* NILs appeared to increase by final harvest, particularly in 2003 (Fig. 1). The increase in total root length in the *tin* NILs reflected increasingly greater root branching (Fig. 1; Supplementary Fig. S1) and commonly increased seminal root length, while seminal root numbers were similar among NILs (Table 1). The biculm B++ tended to have greater total root length and to a lesser extent branch root length than oligoculm B+ in the warm environment (Fig. 1; Supplementary Fig. S1), while both Banks *tin*-containing NILs were similar in the cool, outdoor environment. Nodal root length differences were small until the final harvest, and were typically greater for the free-tillering NILs in all environments (data not shown). Root biomass increased for the *tin*-containing NILs and, together with the small reduction in shoot biomass, contributed to significantly greater root-to-shoot ratios in *tin*-containing NILs (Fig. 1; Table 1; Supplementary Fig. S1). Interestingly, the greater root-to-shoot ratios of the reduced-tillering lines was observed from very early stages of sampling (two leaves onward) and was maintained across the different environments.

Despite the fewer sampling dates for the Silverstar *tin* NILs, significant differences in mean root lengths of 2825cm and 2469cm, and root-to-shoot ratios of 0.29 and 0.19 were observed for Silverstar *tin* and non-*tin* NILs, respectively, at the 7 weeks sampling date in the cooler soil temperatures. This difference was maintained at warmer temperatures, with root lengths of 2020cm and 1634cm, and root-to-shoot ratios of 0.25 and 0.21, for *tin* and non-*tin* NILs, respectively.

Across sampling dates and NILs in all experiments, genotypic increases in total root biomass were closely associated with increases in total root length (*r*_g_=0.99, *P*<0.01) and root branching (*r*_g_=0.99, *P*<0.01), while total root length and branching were themselves strongly correlated (*r*_g_=0.98, *P*<0.01). Genotypic differences in total seminal root length were strongly correlated with total root weight (*r*_g_=0.92, *P*<0.01) and branch root length (*r*_g_=0.78, *P*<0.01) but only moderately correlated with total root length (*r*_g_=0.64, *P*<0.01).

### De-tillering study

**Fig. 2.**
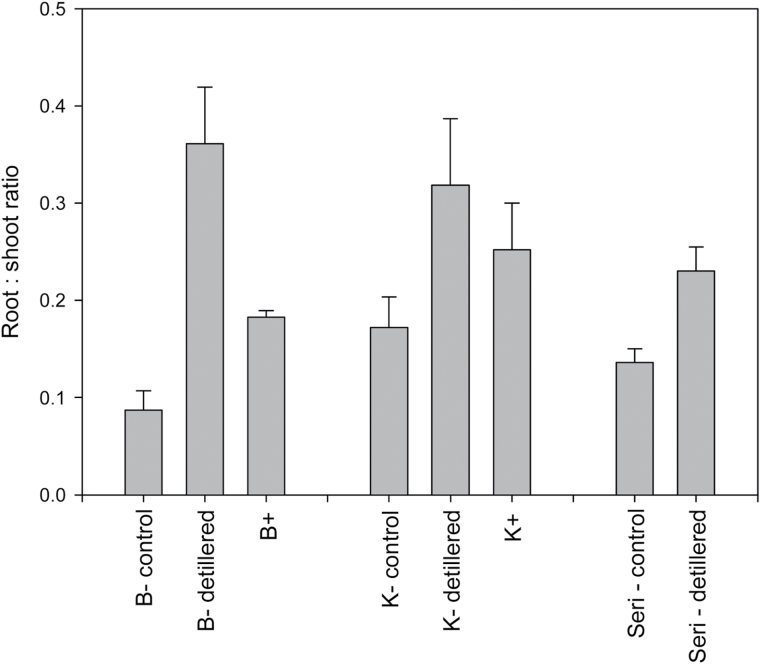
Root-to-shoot ratio at anthesis for three sets of lines including the free-tillering control, a detillering (‘detillered’) treatment and/or a near-isogenic (NIL) pair (B = Banks, K = Kite) containing the *tin* (‘+’) reduced-tillering gene and its non-*tin* (‘–‘) sister NIL. Standard error bars are given.

**Fig. 3.**
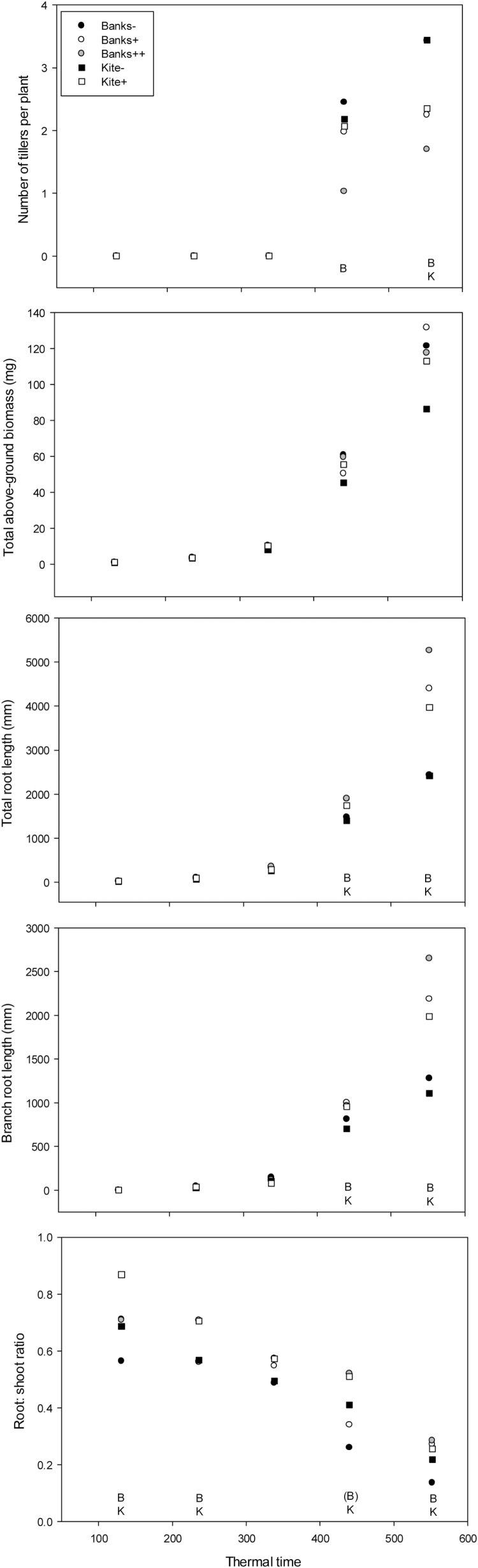
Shoot and root growth for Banks (circlels) and Kite (squares) tillering NILs (open symbol=+*tin* allele except Banks where the shaded circle is biculm expression) assessed over multiple sampling dates in the field in 2004. ‘B’ and ‘K’ denote statistical difference between the Banks and Kite *tin* and non-*tin* NILs, respectively, at *P*=0.05.

The free-tillering, *tin*, and de-tillered non-*tin* treatments averaged 4.7, 1.2, and 1.0 spikes per plant at anthesis, respectively. The continued removal of tillers was associated with a highly significant (*P*<0.01) reduction in numbers of spikes (–79%) and total shoot dry matter (–59%), greater individual stem weight (+36%), and a small but significant increase in root biomass (+35%) and increased root-to-shoot ratio (+232%) in de-tillered plants (Fig. 2). Similarly, increases in the root-to-shoot ratio were significantly greater for *tin*-containing Banks and Kite NILs, consistent with glasshouse and field experiments (compare Figs 1–3; Table 1; Supplementary Fig. S1 at *JXB* online). Compared with the influence of the *tin* gene in Banks and Kite, manual de-tillering was associated with significantly smaller shoot and root biomass, but greater root-to-shoot partitioning. Among backgrounds, smaller root-to-shoot partitioning in the Banks background was consistent with earlier *tin* NIL assessments. Across all genotype and de-tillering treatment means (*n*=8), increases in tiller number were negatively correlated (*r*_p_= –0.93, *P*<0.01) with reductions in the root-to-shoot ratio.

### Field validation

The Bethungra field season was favourable for growth with good January–April, pre-sowing rainfall (70mm) and good in-crop rainfall through to maturity (375mm), consistent with expectation at this site. The 10 month fallow period prior to sowing together with the in-season rainfall ensured the profile was wet to depth, facilitating potential for deep root penetration. Air temperatures were mild, with daily averages of 8.3 °C for the June–September period up to anthesis, and 15.5 °C for the October–December grain-filling period. Averaged across the free-tillering Banks and Kite genotypes, grain yield was high (6.1 t ha^–1^), reflecting large average grain numbers (20 519 m^–2^) but smaller average grain size (30mg) (Table 4).

#### Tiller, spike and leaf area development

Plant counts made soon after seedling emergence indicated no differences in the number of plants m^–2^ between individual lines (data not shown). The first tillers appeared at ~3.3 leaves, but differences in tiller number per plant were not apparent until the fourth harvest at ~450 °Cd (i.e. fourth leaf stage) (Fig. 3; Table 2). The Banks *tin* lines produced significantly fewer tillers per plant than B–, and the biculm B++ NIL produced significantly fewer tillers than the oligoculm B+. Differences in tillering in the Kite background were less pronounced owing to increased production of coleoptile tillers in the K+ plants (data not shown). By the fifth harvest (72 DAS, average leaf stage 5.2), both Banks and Kite *tin* lines produced significantly fewer tillers than non-*tin* sister lines (–43% and 37% for Banks and Kite, respectively, at 560 °Cd; Fig. 3.). Reductions in tillering were associated with significantly smaller shoot biomass for the B++ but not B+ NIL, and no difference in the biomass of the Kite NILs (Table 2).

**Table 2.** Shoot and root characteristics for Banks and Kite tillering NILs at 58 DAS for field-assessed plants in 2004

Entry	Shoot biomass (mg)	No. of tillers per plant (*n*)	Total root biomass (mg)	Total root length (mm)	Branch root length (mm)	Seminal root length (mm)	No. of seminal roots (*n*)	No. of nodal roots (*n*)	Root: shoot ratio	Root proportion^*a*^
Banks–	60.6	2.5	15.6	1477	838	637	4.32	4.22	0.26	0.20
Banks+	69.6	1.9*	24.0**	2053**	1061**	991**	4.89**	5.14**	0.34*	0.26*
Banks++	45.1**	1.0**	23.3**	1903**	965*	939**	4.83**	5.09**	0.52**	0.34**
Kite–	43.4	2.2	17.9	1353	681	673	4.65	4.00	0.41	0.29
Kite+	56.0	2.0	28.5**	2007**	1079*	929*	5.11**	4.44**	0.51*	0.34

^*a*^ Proportion of total plant biomass that is root biomass

* and ** denote that restricted-tillering, *tin* NIL means are statistically different from non-*tin*, free-tillering sister NIL means at *P*=0.05 and 0.01, respectively. –, +, ++ refer to free tillering, oligoculm and biculm NILs, respectively.

Early leaf area and plant biomass were the same for all lines (Fig. 3). However, reduced tillering in the Banks *tin* NILs contributed to significant reductions in anthesis LAI (–40%), light interception (–12%), and biomass (–22%) (Table 3). This reduction was particularly large for B++ (e.g. 49% for LAI), whereas the reduced tillering in B+ contributed to significantly reduced LAI and light interception but not biomass. The Kite NILs were not statistically different for LAI and light interception at anthesis, but the *tin*-containing K+ NIL was reduced for biomass. At low sowing densities, reduced-tillering Banks and Kite lines produced significantly less anthesis biomass than their free-tillering sister NILs (Table 3). This reduction was relatively greater than for anthesis biomass at normal sowing density, with reductions in biomass of all *tin*-containing NILs of 19% and 33% at normal and low plant density treatments, respectively (Table 3). Increased light interception across all genotypes was significantly correlated with changes in anthesis biomass at normal (*r*_p_=0.95, *P*<0.05) and low (*r*_p_=0.95, *P*<0.05) plant densities, while anthesis biomass at low and normal plant densities was itself strongly correlated across genotypes (*r*_p_=0.96, *P*<0.05). Stem nitrogen concentration was greater for B++ but not B+, while leaf N concentration was greater for *tin* NILs in the Banks but not the Kite genetic backgrounds.

**Table 3.** Leaf area index, light interception, biomass (including low density), and stem and leaf nitrogen concentration at anthesis for Banks and Kite tillering NILs assessed in the field in 2004

Entry	Leaf area index (m^2^ m^–2^)	Light interception (%)	Anthesis biomass (normal density) (g m^–2^)	Anthesis biomass (low density) (g m^–2^)	Stem nitrogen concentration (%)	Leaf nitrogen concentration (%)
Banks–	4.22	92	888	979	0.65	2.70
Banks+	2.92**	85**	809*	649**	0.66	3.08**
Banks++	2.11***	77***	576***	449***	0.81***	2.99*
Kite–	5.94	94	1095	1129	0.68	2.59
Kite+	5.49	93	919*	869**	0.59	2.65

*, **, and *** denote that restricted-tillering, *tin* NIL means are statistically different from non-*tin*, free-tillering sister NIL means at *P*=0.10, 0.05, and 0.01, respectively. –, +, ++ refer to free tillering, oligoculm and biculm NILs, respectively.

#### Root growth

Differences in root growth in the field mirrored observations for these same lines under controlled-environment conditions. Total root length was the same for all lines until early tillering, after which the *tin* NILs produced significantly greater total root length (Fig. 3) and biomass (Table 2). Greater total root length reflected significant differences in seminal root length and extent of root branching between *tin* and non-*tin* NILs, and were maintained from tillering to final harvest at 72 DAS. By the last harvest, the *tin*-containing NILs produced approximately double the branched and total root length of the non-*tin* NILs. During the early stages of plant growth, *tin*-containing NILs produced significantly greater numbers of seminal roots but there was no difference in seminal root number between the B+ and B++ NILs (Table 2). Seminal root length varied significantly between *tin* and non-*tin* NILs, with the longest B–, B+, and B++ NILs averaging seminal root lengths of 146, 202, and 195cm, respectively. Nodal roots were recorded and were significantly greater in number for *tin* lines (Table 2). Only the Banks NILs were cored and measured for root depth at maturity. Here the *tin*-containing B+ and B++ NILs were significantly deeper at average depths of 147cm and 135cm, respectively, compared with 117cm for the non-*tin* B–NIL (Table 4). For all sample dates, the root-to-shoot ratio was significantly greater in the *tin*-containing NILs, and, as for the controlled-environment studies, the root-to-shoot ratio decreased progressively from sowing (Fig. 3).

**Table 4.** Means for agronomic traits measured on Banks and Kite tillering NILs assessed during grain-filling and maturity in the field in 2004

Entry	Plant height (cm)	Stomatal conductance^*a*^ (*n*)	Canopy temperature depression^*a*^ (°C)	No. of spikes (*n* m^–2^)	Grain yield (t ha^–1^)	Total biomass (t ha^–1^)	Harvest index	No. of grains (*n* m^–2^)	Grains per ear (*n*)	Kernel weight (mg)	Grain protein concentration (%)	Root depth (cm)
Banks–	100	0.13	0	608	5.91	14.5	0.41	22 692	38	26	11.8	117
Banks+	97	0.29**	–0.41	328***	6.90**	15.0	0.45**	19 714*	60***	35***	13.1**	147**
Banks++	96	0.38***	–1.40**	237***	6.59**	14.1	0.46***	18 333**	79***	36***	14.5***	135*
Kite–	102	0.14	–^*b*^	536	6.22	15.9	0.39	18 353	34	34	14.5	–^*b*^
Kite+	103	0.16	–^*b*^	447*	6.19	16.3	0.38	14 420**	33	43***	14.6	–^*b*^

^*a*^ 7–10 d post-flowering averaged at three times: 9:00, 12:00, and 15:00h.

^*b*^ Not sampled.

*, **, and *** denote that restricted-tillering, *tin* NIL means are statistically different from non-*tin*, free-tillering sister NIL means at *P*=0.10, 0.05, and 0.01, respectively. –, +, ++ refer to free tillering, oligoculm and biculm NILs, respectively.

#### Agronomic performance and water use

Differences in total tiller number increased with time, so that by maturity the B++ and B+ NILs produced 61% and 41% fewer spikes, respectively, than B– (Table 2). The difference in the Kite background was smaller, with a reduction of ~20% spikes for K+. Grain yields were not different between genetic backgrounds, averaging 6.47 t ha^–1^ and 6.20 t ha^–1^ for Banks and Kite, respectively (Table 4). Within backgrounds, the *tin*-containing Banks NILs produced significantly greater yield, whereas the Kite NILs were not different. The greater yield for *tin* in the Banks background was associated with a significant increase in harvest index, whereas total biomass was not different. Harvest index and biomass were the same for B++ and B+ NILs. Numbers of spikes and grains per m^2^ was commonly fewer, but numbers of grains per spike were greater for *tin*-containing Banks NILs. Reductions in numbers of grains were compensated by significant increases in average kernel weight. The Kite *tin* NILs produced significantly fewer spikes and fewer kernels of larger average size, whereas grains per spike were the same for both Kite NILs (Table 4). The B+ and B++ NILs were significantly greater for grain protein concentration and content than B–, whereas K– and K+ NILs were no different. The B++ NIL was significantly greater for grain protein concentration and content than B+.

**Fig. 4.**
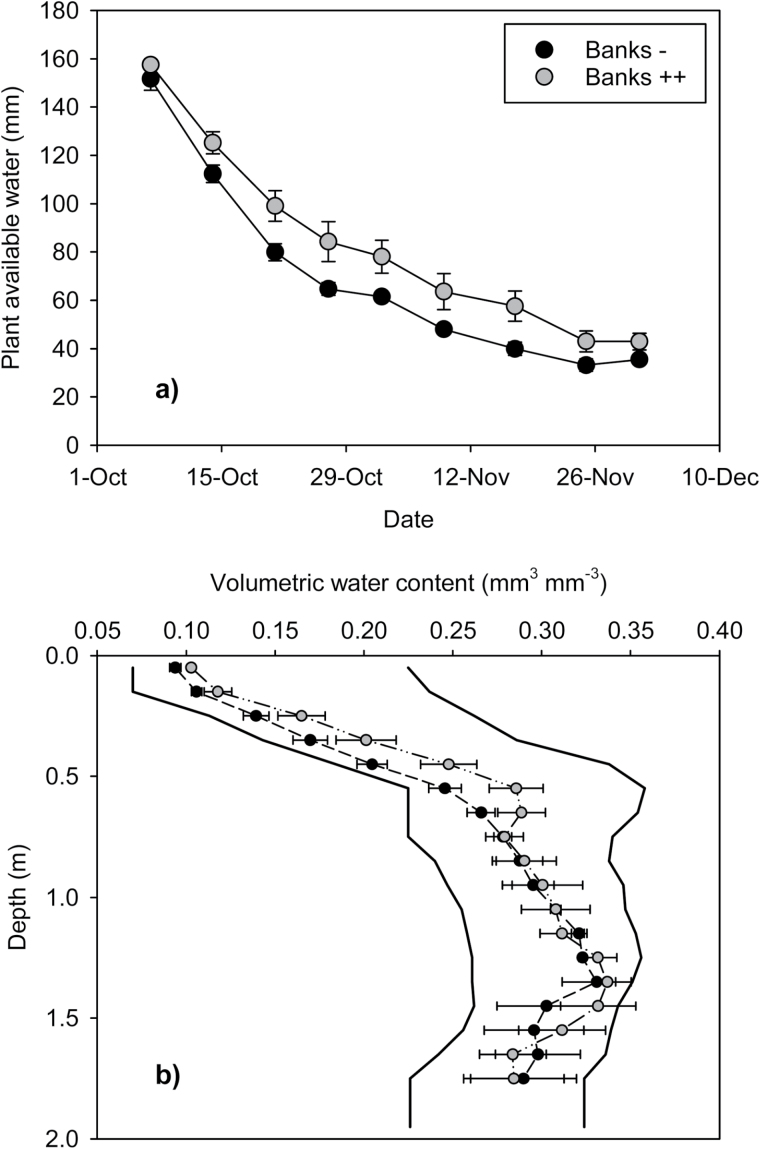
(a) Change in plant-available water in the soil (0–1.4 m) for two wheat Banks NILs (Banks– and Banks++) from the wettest profile around anthesis (7 October) until maturity (2 December) showing slower water use and higher soil water levels under Banks++, and (b) the profile of soil water on 21 October (point of maximum difference) for Banks– and Banks++. The solid lines in (b) show the drained upper limit and lower limit of water extraction, and the horizontal bars in both graphs are the SEM.

The *tin*-containing NILs had significantly greater stomatal conductance early in grain-filling (7 d post-anthesis), but only the Banks *tin* NILs maintained high conductance through to physiological maturity (Supplementary Fig. S2 at *JXB* online). The greater conductance and thereby transpiration of B+ and B++ translated into significantly cooler and subsequently greater canopy temperature depression (CTD) for these NILs (Table 4). The *tin*-containing Banks NILs maintained significantly greater green leaf number during grain filling, particularly B++ with an average one green leaf per stem at physiological maturity (Supplementary Fig. S3). Despite the over-riding influence of *tin* in the Banks NILs, stomatal conductance, CTD, and green leaf number through grain filling were consistently greater for the biculm B++ than the oligoculm B+. Separate from improved root growth, water use was slowed by the presence of *tin*, and this was especially obvious during the post-anthesis drying period when water use patterns were less obscured by the frequent rainfall (Fig. 4a). From early October when the profiles in both B– and B++ had been filled, and total plant available water (PAW) was similar, water use was slower in B++ which preserved higher levels of PAW than B– throughout most of the grain-filling period (Fig. 4a). The increased soil water was present in the upper soil layers, with little significant difference below 1 m at least during mid-grain-fill (Fig. 4b). By maturity, most of the PAW had been used in both B– and B++ (Fig. 4a), but the deferred water use and higher soil water levels in B++ presumably served to maintain higher conductance and green leaf area during grain-fill.

## Discussion

The Passioura identity (1977) defines grain yield in water-limited environments as integrating three physiological components: total water use and water use efficiency in building crop biomass, and then harvest index in final determination of yield. In wheat, robust and repeatable genetic variation has been established for traits contributing to greater water use efficiency (e.g. Richards *et al.*, 2002) and harvest index (Duggan *et al.*, 2005a; Rebetzke *et al.*, 2008*a*), while reduced heritability has limited the reliable identification of genetic variation for factors such as root architecture and size affecting capacity for greater water use. The potential to modify root morphology and growth (e.g. changes in root angle, diameter, and branching, root number, and root depth; e.g. Christopher *et al.*, 2013) requires confirmation in the field (e.g. Wasson *et al.*, 2012), while separate studies are required to establish related changes in water uptake, particularly deeper in the soil profile (Lopes and Reynolds, 2010). Validation of complex phenotypes such as shoot and root growth requires translation to the field before uptake and use in breeding programmes (Rebetzke *et al.*, 2014). The studies reported herein summarize a continuum of activities extending from glasshouse to field, targeting assessment of the effects of the *tin* gene on root and shoot growth, and their impacts on the components of the Passioura identity.

### Root growth

The current study has confirmed earlier observations (Duggan *et al.*, 2005b) of the influence of a major tiller inhibition gene on partitioning of carbon to roots to increase post-tillering root biomass through increased root branching and subsequently greater total root length. The *tin* gene was associated with a longer, more branched, and a heavier root system from early in plant development, and this effect was consistent in controlled glasshouse and field studies, and, to a lesser extent, across genetic backgrounds. The increased root growth translated into deeper roots in the field and was greatest in cooler soils. Numbers and sizes of seminal roots were generally not different between *tin* and non-*tin* lines, while differences in nodal root length and biomass were commonly small. Together, the *tin* gene appeared to increase the number of first- and second-order laterals on the seminal axes.

Root growth was generally similar for all lines following emergence and during early seedling growth. Changes in root size between lines coincided with differentiation in tillering. The extent of root growth was approximately the same for oligoculm and biculm Banks NILs, while even small reductions in tillering in the Kite *tin* NIL was associated with greater root growth. The main root components changing with *tin* were seminal root lengths and their branches. Seminal root number was the same for *tin* in the controlled environments but was greater for *tin* wheats in the field study. Branch roots are finer and reportedly more effective in absorbing water and nutrients (Mackey, 1986), while a greater frequency of longer seminal roots presumably allows for greater exploration of the soil, particularly deeper soil layers, if water is available later in the season (Kirkegaard *et al.*, 2007; Wasson *et al.*, 2012). Root biomass was increased relative to shoot biomass in *tin*-containing wheats. Root-to-shoot ratios were also increased across multiple genetic backgrounds following manual de-tillering, and the size of the increase was consistent with the influence of *tin* on the root-to-shoot ratio in the same studies.

The greater root-to-shoot ratio of *tin* lines might be explained by a change in carbohydrate allocation to roots. An increased root-to-shoot ratio was very apparent at commencement of tillering. From this it was hypothesized that carbohydrates normally allocated to developing tillers were instead allocated to roots. Both genetic and management-like factors influencing spike size and/or number have demonstrated changes in shoot carbon to affect the root-to-shoot ratio and root length directly. These studies have included simple ear removal from before or at anthesis in both wheat and barley, and the monitoring of carbohydrate allocation from ears to roots to increase root biomass (e.g. Austin and Edrich, 1975; Koide and Ishihari, 1992). Other ear removal studies aimed at removal of competing sinks confirm a ubiquitous partitioning of carbon to other organs including roots (e.g. King *et al.*, 1967; Gu and Marshall, 1988; Sharma-Natu and Ghildiyal, 1994). Early tiller removal or defoliation resulted in increased allocation of carbon to retained shoots to increase stem and leaf sheath thickness, increase leaf area, and reduce SLA (Gu and Marshall, 1988). These phenotypes are consistent with the *tin* phenotype observed for the Banks, Kite, and Silverstar backgrounds reported herein. In a separate genetics experiment, divergent selection into groups of related wheat lines contrasting for numbers of spikes at maturity was associated with increased water extraction below 90cm for the low spike number-selected group (Innes *et al.*, 1981). It is unlikely that lines in that study varied for presence of the *tin* gene, but the observations confirm a resource allocation link between fewer spikes and the potential for more effective rooting at depth.

### Shoot growth

Growing conditions early in the season are often conducive to the initiation and development of many tillers to produce large leaf areas and biomass (Berry *et al.*, 2003; Duggan *et al.*, 2005a; Rebetzke *et al.*, 2008*b*; Mitchell *et al.*, 2013; Moeller *et al.*, 2014). In the current study, shoot growth in seedlings was similar for *tin* and non-*tin* NILs, with reductions in leaf area and biomass not apparent in *tin* NILs until some time after commencement of tillering, and then more so in the extreme biculm *tin* types. Continued suppression of tillering resulted in generally reduced LAI and light interception, and reduced biomass at anthesis particularly at low plant densities where a lack of plasticity in the *tin*-containing NILs limited their ability to respond with more tillers and ultimately more spikes (Sadras and Rebetzke, 2013).

The influence of *tin* on tiller reduction was dependent on the genetic background and the thermal environment under assessment. Selection for fewer tillers in development of the B++ genotype was associated with reliably fewer tillers and final spike number, whereas the intermediate tillering B+ produced fewer tillers than the wild-type B– but was rarely as extreme in tiller reduction as its B++ biculm sibling. Similarly, with the *tin* gene in the Silverstar background, tillering was reliably less whereas *tin* expression in the Kite background was more variable in both cool and warm environments. Tiller expression in the cooler field experiment generally mirrored expression under cool controlled environment conditions. That is, differences in *tin* expression were maintained in the Banks backgrounds whereas tiller reduction was smaller in the Kite background, consistent with glasshouse assessments. Nonetheless, Kite *tin* NILs ultimately produced significantly fewer spikes in the field, consistent with the Banks B+ NIL. Selection for extreme reduction in tillering in B++ appears to have favoured a reliably extreme restricted *tin* tillering genotype, whereas selection for a more free-tillering *tin* genotype has produced NILs which are more like the non-*tin*, free-tillering NIL particularly early in the season. This was the case for both B+ and K+ NILs where differences in tiller and spike number were obvious from stem elongation onward, and particularly at anthesis.

Several studies have reported the influence of tillering on growth and yield of wheat in well-watered and water-limited environments alike. More recent studies have included assessment of the *tin* gene on reductions in tillering and final spike number in low-yielding environments (e.g. Duggan *et al.*, 2005a; Mitchell *et al.*, 2013; Moeller *et al.*, 2014), and focusing on tiller development and correlated changes in tiller number, leaf area, and water use. Few studies have reported the influence of *tin* in favourable environments, although Gaju *et al*. (2009, 2014) and Mitchell *et al.* (2013) demonstrated small yield reductions for *tin*-containing recombinant inbred lines/double haploid lines under irrigation. The studies herein confirm other studies reporting the presence of *tin* reduced tillering in both controlled and a range of field environments in showing that fewer tillers translated into reductions in final spike number. Leaf area and anthesis biomass were also reduced. However, as reported here, the extent and timing of tiller inhibition with *tin* varied with genetic background, as did the magnitude of the impact on anthesis biomass and final spike number.

Previous studies have demonstrated the presence of the *tin* gene to be associated with changes in a number of other shoot characteristics (Duggan *et al.*, 2005a). Along with tiller size, biomass, and leaf area, the SLA and stem size also increased. Changes in tillering mirrored changes in total leaf area and biomass, light interception, and anthesis biomass. The above-ground *tin* phenotypes observed in this study were consistent with the previous reports (e.g. Duggan *et al.*, 2005a; Mitchell *et al.*, 2012) and included reduced tiller and spike density; a higher proportion of productive tillers; thicker leaves and reduced SLA; a small reduction in above-ground biomass; increased numbers of kernels per spike; and larger average kernel size. In irrigated, high radiation environments aimed at very high yield potentials, Gaju *et al.* (2014) reported that the *tin* gene was associated with increased light interception from stem elongation to anthesis but reduced radiation use efficiency. In their study, high grain yields for *tin*-containing lines reflected increased carbon partitioning to spikes to increase grain number per spike and unit area despite significant reductions in spike number.

### Links between modified root and shoot growth, water use, and grain yield in water-limited environments

The impact of any change in the root and shoot characteristics of plants on crop yield is ultimately determined by the specific pattern of water availability throughout the season, its impact on the timing and severity of water stress, and how these interact with the development and realization of season-specific yield potential. Our central hypothesis was that in storage-driven, agro-environments subject to increasing terminal stress, deferred water use from pre- to post-anthesis could be beneficial to yield. The *tin* gene offered opportunities to achieve this by simultaneously restricting pre-anthesis biomass growth (and hence water use) while increasing the capacity of the root systems to better exploit soil water during the post-anthesis phase through a deeper and more branched root system (e.g. Lopes and Reynolds, 2010). The extent to which links between modified root and shoot growth, water use, and grain yield can be demonstrated in field studies is confounded by the inherent variability in seasonal conditions, a fact that leads many to manage environments with irrigation and rain-out shelters (Mitchell *et al.*, 2013; Rebetzke *et al.*, 2013a) and/or supplement experimental studies with calibrated crop simulation models that capture seasonal variability. Lilley and Kirkegaard (2007) clearly showed how seasonal variability generated a wide range of expected benefit of deep soil water to wheat crops which averaged 37kg grain ha^–1^ mm^–1^, but ranged from 0 to 60kg ha^–1^ mm^–1^ according to seasonal rainfall distribution.

The seasonal conditions experienced in the field study reported herein did not feature the severe terminal stress commonly envisaged for storage-driven systems (e.g. Mitchell *et al.*, 2013), but nonetheless evidence was provided that supported several aspects of the central hypothesis. The reduction in tillering and biomass growth up to anthesis was associated with conservation of soil water during the post-anthesis period (Fig. 4a, b). The reduction in tillering and biomass did not come at the expense of reduced yield potential, at least up to the high ~6.0 t ha^–1^ potential achieved at the site, because the *tin*-containing lines were able to compensate for reduced spike number by significantly increasing the numbers of grain per spike. As a result, yield potential (grain number) was not compromised, while water was preserved to assist the crop in filling the grains that had been set. The capacity to fill the grains was facilitated by two processes: first, the increased residual soil water available after anthesis which maintained green leaf area and photosynthesis (Supplementary Figs S2, S3 at *JXB* online), and the deeper and more branched root system increased the likelihood that photosynthesis could be maintained later into grain-filling to ensure larger grain; secondly, a feature of *tin*-containing lines is their higher levels of stem-soluble carbohydrates with capacity to remobilize these should current photosynthesis be restricted by drying soils (Rebetzke *et al.*, 2008*a*; Mitchell *et al.*, 2013). Though much of this second process is expressed under more severe terminal stress than those experienced here (e.g. van Herwaarden *et al.*, 1998; Rebetzke *et al.*, 2008*a*), previous experiments conducted under conditions of less water-limiting post-anthesis conditions similar to those experienced in this experiment identified *tin* lines that achieved relatively high grain yield and maintained a kernel number and weight advantage (e.g. Mitchell *et al.*, 2012; Gaju *et al.*, 2014). In fact, compensation for the decrease in spike density can result in similar or even higher grain yields for reduced-tillering *tin* lines (A.F. van Herwaarden, unpublished data). In some situations, even uniculm wheats have been reported to be higher yielding than free-tillering sister lines (Islam and Sedgley, 1981). Whan *et al.* (1988) also observed that reduced-tillering lines compensate for fewer numbers of spikes by increasing kernel number per ear and/or production of heavier kernels. Increased grain yield does not appear to be due to the *tin* gene itself but reflects the reduced-tillering response associated with this gene.

The soil water profiles at harvest were similar for the *tin* and non-*tin* lines so it appears that the deeper and more branched root system did not play a major role in the maintenance of green leaf area, and grain-filling in the *tin* lines. Rather the slower and deferred water use due to reduced shoot biomass prolonged green leaf duration and assimilation, and, together with the redistribution of soluble carbohydrates, facilitated grain-filling. We cannot be certain whether the increased grain nitrogen reflected increased N uptake with deeper, more extensive roots. Nevertheless, numerous experimental and simulation studies have demonstrated the benefits of a deeper and more effective root system in providing the potential to support higher yield through access to water late in the season (e.g. Lopes and Reynolds, 2010). Our study demonstrates that *tin*-containing lines can achieve deeper and more branched roots without the often assumed compromising of shoot growth and yield potential. Though not expressed under the seasonal conditions experienced here, the deeper and more extensively branched root system will inevitably benefit crop growth under conditions of more severe terminal stress than those experienced here (e.g. Mitchell *et al.*, 2013).

It is well established that the *tin* gene has a major effect on tiller suppression and biomass accumulation but that tiller expression varies both between and within genetic backgrounds (Mitchell *et al.*, 2013; Gaju *et al.*, 2014). Herein, there was a 2-fold range in tiller number between B+ and B++ NILs in controlled and field studies. Similarly, the effect of *tin* on root growth and subsequent root-to-shoot ratios varied depending on background and somewhat independently of tiller number. Despite evidence for an association between *tin* and increased root growth, other factors appear to contribute to root growth (e.g. Bai *et al.*, 2013; Liu *et al.*, 2013), suggesting the need for careful assessment within *tin*-varying populations to target increased root growth.

## Conclusions

The current study has demonstrated that the presence of the *tin* gene and its likely role in modifying tiller number has slowed and deferred water use, while maintaining yield potential (grain number), and contributed to changes in seminal and branch root length to increase total root length and biomass. The influence of *tin* in changing plant carbon dynamics is supported by de-tillering studies demonstrating altered carbon movement to increase root growth. Coupled with previous observations of improved water extraction with reduced tillering in *tin* (Mitchell *et al.*, 2013) and non-*tin* (Innes *et al.*, 1981) studies elsewhere, validation in the field confirms maintenance of transpiration and green leaf area, and changes in shoot and root growth combine to maintain favourable conditions for grain growth during grain-filling.

## Supplementary data

Supplementary data are available at *JXB* online.

Figure S1. Shoot and root biomass for Banks and Kite tillering NILs assessed over multiple sampling dates in 2004 in cool outdoor and warm glasshouse conditions.

Figure S2. Mean leaf conductance (as 1/leaf porosity) scores for three dates post-anthesis for Banks and Kite tillering NILs.

Figure S3. Numbers of green leaves per spike estimated during and after mid grain-filling for the Banks tillering NILs evaluated in the field in 2004.

Supplementary Data

## Abbreviations:

NIL: near-isogenic line
SLA: specific leaf area
tin: tiller inhibition.
