# Supplementary material for: A tillering inhibition gene influences root–shoot carbon partitioning and pattern of water use to improve wheat productivity in rainfed environments
Source: J Exp Bot. 2015 Oct 22;67(1):327–40. doi: 10.1093/jxb/erv457 (PMC4682434; doi:10.1093/jxb/erv457)
Supplement: Supplementary Data [file supp_67_1_327__index.html]

A tillering inhibition gene influences root–shoot carbon partitioning and pattern of water use to improve wheat productivity in rainfed environments — A tillering inhibition gene influences root–shoot carbon partitioning and pattern of water use to improve wheat productivity in rainfed environments — Supplementary Data 

# A tillering inhibition gene influences root–shoot carbon partitioning and pattern of water use to improve wheat productivity in rainfed environments

## Supplementary Data

Data files

- Supplementary Data - Supplementary Data
