# Supplementary material for: A tillering inhibition gene influences root–shoot carbon partitioning and pattern of water use to improve wheat productivity in rainfed environments
Source: J Exp Bot. 2015 Oct 22;67(1):327–40. doi: 10.1093/jxb/erv457 (PMC4682434; doi:10.1093/jxb/erv457)
Supplement: Supplementary Data [file supp_erv457_Supplementary_figures.pdf]

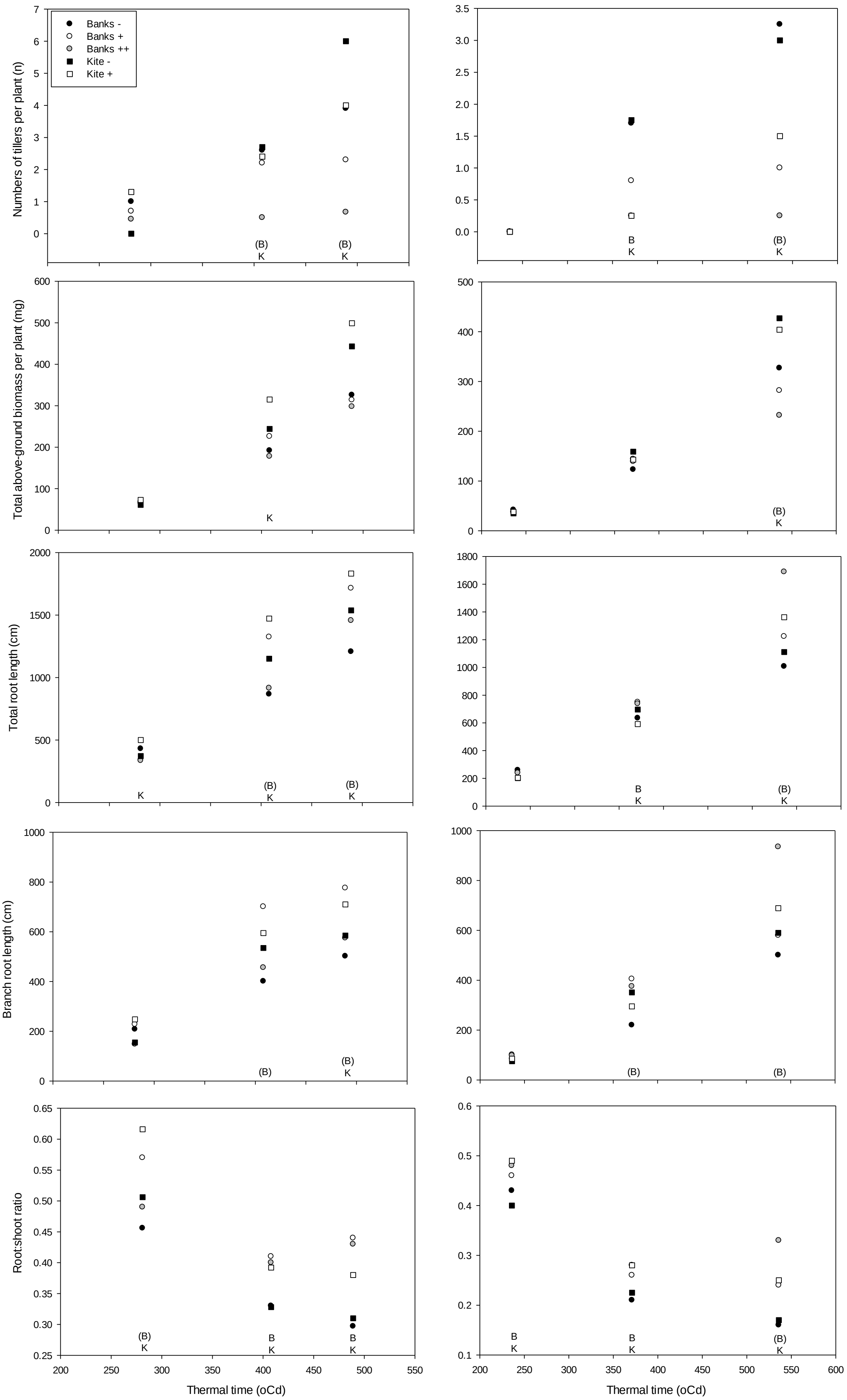

Banks -
Banks +
Banks ++
Kite -
Kite +
Numbers of tillers per plant (n)
Total above-ground biomass per plant (mg)
Total root length (cm)
Branch root length (cm)
Root:shoot ratio
Thermal time (oCd)
Thermal time (oCd)

Supp 1. Shoot and root biomass for Banks (○) and Kite (□) tillering NILs (open symbol = +*tin* allele) assessed over multiple sampling dates in 2004 in (a) cool, outdoor and (b) warm glasshouses conditions, respectively. 'B' and 'K' denote statistical difference between the Banks and Kite *tin* and non-*tin* NILs, respectively, and '(B)' indicates biculm and oligoculm *tin*-containing Banks NILs are statistically different at P = 0.05.

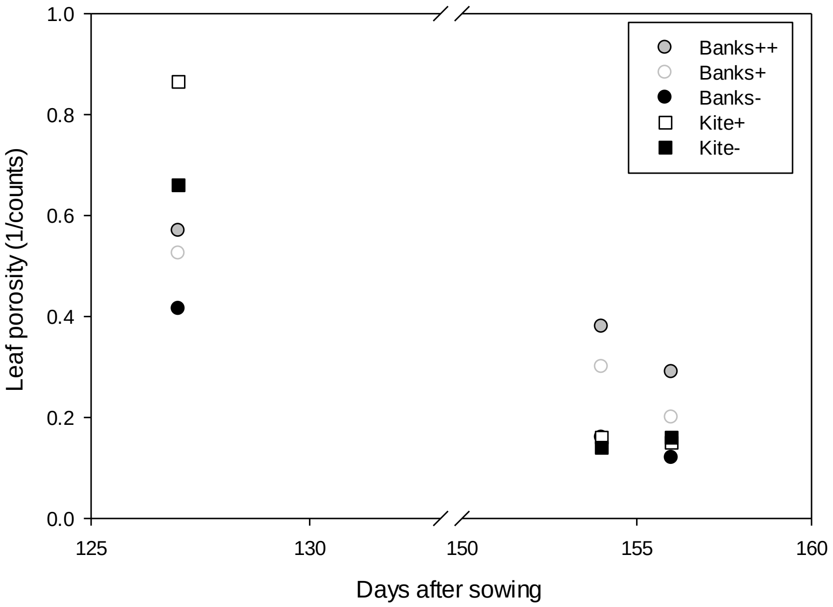

Supp 2. Mean leaf conductance (as 1/leaf porosity) scores for three dates post-anthesis for Banks and Kite tillering NILs. Scores are means for three times on each date. The LSDs for comparing among lines were 0.09, 0.09 and 0.07 for 127, 154 and 156 DAS, respectively.

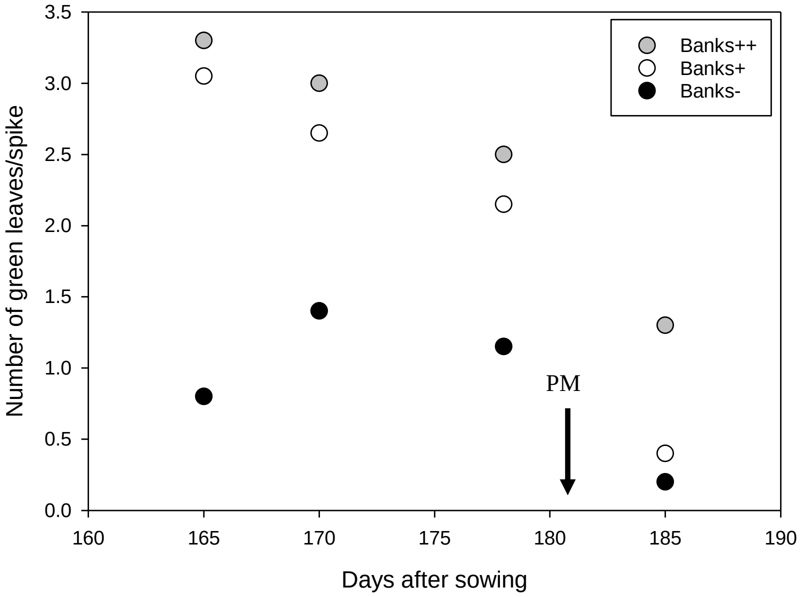

Supp 3. Numbers of green leaves per spike estimated during and after mid grain-filling for the Banks tillering NILs evaluated in the field in 2004. 'PM' nenotes physiological maturity. The LSD for the sample date × NIL interaction was 0.3.
